# Nutritional Status, Uremic Toxins, and Metabo-Inflammatory Biomarkers as Predictors of Two-Year Cardiovascular Mortality in Dialysis Patients: A Prospective Study

**DOI:** 10.3390/nu17061043

**Published:** 2025-03-16

**Authors:** Sylwia Czaja-Stolc, Marta Potrykus, Jakub Ruszkowski, Alicja Dębska-Ślizień, Sylwia Małgorzewicz

**Affiliations:** 1 Department of Clinical Nutrition and Dietetics, Medical University of Gdansk, 80-211 Gdansk, Poland; sylwia.czaja-stolc@gumed.edu.pl (S.C.-S.); sylwia.malgorzewicz@gumed.edu.pl (S.M.); 2 Department of Oncological, Transplant, and General Surgery, Medical University of Gdansk, 80-214 Gdansk, Poland; 3 Department of Nephrology, Transplantology and Internal Medicine, Medical University of Gdansk, 80-214 Gdansk, Poland; jakub.ruszkowski@gumed.edu.pl (J.R.); alicja.debska-slizien@gumed.edu.pl (A.D.-Ś.)

**Keywords:** chronic kidney disease, cardiovascular mortality, adipokines, myokines, gut-microbiota-derived uremic toxins, nutritional status, kidney replacement therapy

## Abstract

Patients with chronic kidney disease (CKD) are at a significantly increased risk of cardiovascular (CV) mortality, which cannot be fully accounted for by traditional risk factors. **Background/Objectives**: The aim of this study is to evaluate the impact of adipokines, myokines, gut-microbiota-derived uremic toxins, and nutritional status on the risk of CV mortality in patients undergoing kidney replacement therapy (KRT). **Methods**: This study includes 84 hemodialysis (HD) patients and 44 peritoneal dialysis (PD) patients. Adipokines and myokines concentrations were measured using enzyme-linked immunosorbent assays (ELISA), while gut-microbiota-derived uremic toxins were quantified using liquid chromatography-tandem mass spectrometry (LC–MS/MS). Nutritional status was assessed using the seven-point Subjective Global Assessment (SGA) and anthropometric measurements. The survival was analyzed using Kaplan–Meier curves with the log-rank test, along with univariate and multivariate Cox proportional hazards regression. **Results**: The mean follow-up period was 18.2 (8) months for the HD group and 14.3 (8) months for the PD group. During the 2-year follow-up, 15.5% of HD patients and 6.8% of PD patients died due to cardiovascular disease (CVD). In the HD group, age, blood urea nitrogen (BUN), phosphorus, interleukin-6 (IL-6), high-sensitivity *C*-protein (hsCRP), and neutrophil-to-lymphocyte ratio (NLR) levels were significantly associated with CV mortality. HD patients who died had significantly lower myostatin/IL-6 ratios. CV mortality was significantly associated with age and potassium levels in the PD group. **Conclusions**: The examined adipokines, myokines, and gut-microbiota-derived uremic toxins exert a less significant direct influence on survival compared to widely recognized indicators, including age, nutritional status, and inflammatory markers.

## 1. Introduction

Approximately 4 million people worldwide undergo kidney replacement therapy (KRT), with hemodialysis (HD) being the most commonly used method. It accounts for around 70% of all KRT and approximately 90% of all dialysis treatments [1]. In Poland, an estimated 20,000 patients receive dialysis [2].

Patients with chronic kidney disease (CKD) have a heightened risk of cardiovascular diseases (CVD). This risk is significantly higher in early CKD stages (1–3) compared to the general population and increases further in advanced stages (4–5) [3]. In 2021, CVD was observed in 77.3% of patients undergoing HD, 68.3% of patients on peritoneal dialysis (PD), and 54.5% of kidney transplant recipients (KTR) [4]. CVD is the leading cause of death among patients with CKD.

Traditional cardiovascular risk (CVR) factors, such as smoking, hypertension, carbohydrate metabolism disorders, and dyslipidemia, are highly prevalent in patients with CKD and play a significant role in the development of atherosclerosis. These factors not only increase the risk of cardiovascular (CV) and cerebrovascular events, but also contribute to CKD progression [1]. However, traditional risk factors do not fully explain the increased risk of CVD in patients with CKD [5]. There is a growing interest in the role of gut microbiota, their metabolites, and the nutritional status of patients in this phenomenon. Gut-microbiota-derived uremic toxins, such as trimethylamine-*N*-oxide (TMAO), *p*-cresyl sulfate (pCS), and indoxyl sulfate (IS) have emerged as important contributors to systemic inflammatory and oxidative stress [6,7]. Adipokines, such as leptin, adiponectin, and zinc-alpha2-glycoprotein (ZAG), along with myokines, including irisin and myostatin, are bioactive molecules that facilitate communication between adipose tissue, skeletal muscle, and other organs. These molecules have been implicated in metabolic and inflammatory pathways relevant to CV health [8,9]. While some of the factors mentioned are frequently studied, biomarkers like ZAG and myostatin have been described in only a limited number of publications related to CKD patients. CVR is also influenced by the occurrence of nutritional disorders, both obesity and malnutrition and sarcopenia. Obesity and sarcopenia are closely linked, driven by dysfunctions in adipose tissue and skeletal muscle. CKD is one of the major conditions associated with premature and accelerated aging [10]. Aging promotes adipose inflammation, resulting in fat redistribution to visceral areas and fat infiltration into muscles, which reduces strength and functionality. Lipid accumulation in and around muscle cells disrupts mitochondrial function, increases reactive oxygen species (ROS), and contributes to lipotoxicity, insulin resistance, and pro-inflammatory cytokine production. These muscle-derived cytokines, in turn, can exacerbate adipose tissue dysfunction, perpetuating a cycle of local hyperlipidemia, chronic inflammation, and insulin resistance [11].

Existing research on CVR in patients undergoing KRT often focuses on isolated factors, such as inflammation, oxidative stress, or individual biomarkers. However, there is a significant gap in studies that comprehensively assess the interplay between nutritional status, gut-microbiota-derived uremic toxins, adipokines, myokines, and other nutritional parameters in this context. Given the complexity of the metabolic and inflammatory pathways involved in CVD, broader and more integrative research is essential to uncover potential synergistic effects and provide deeper mechanistic insights.

The objective of this study was to evaluate the influence of adipokines, myokines, gut-microbiota-derived uremic toxins, other biochemical factors, and nutritional status on the risk of CV mortality in patients undergoing KRT.

## 2. Materials and Methods

### 2.1. Study Design

An observational prospective study evaluated the association between adipokines, myokines, gut-microbiota-derived uremic toxins, other biochemical factors, nutritional status, and 2-year CV mortality in patients undergoing KRT. This article constitutes the final part of a comprehensive study. Previous publications have reported findings on adipokines, myokines, nutritional status, and the impact of diet on gut-microbiota-derived uremic toxins. The full methodology of the study was described previously [12,13]. This study was conducted in accordance with the Declaration of Helsinki and was approved by the Independent Bioethical Committee of the Medical University of Gdansk (NKBBN/613/2020, 24 November 2020).

### 2.2. Study Population

The inclusion criteria for the study were as follows: patients over 18 years of age, a minimum of three months on dialysis for HD and PD patients, or at least three months post-transplantation for KTRs, stable clinical condition without recent surgical or infectious complications, including signs of allograft rejection. The exclusion criteria included inability to provide informed consent, cognitive impairment, and active oncological diseases.

Originally, this study included 84 HD patients, 44 PD patients, 52 KTRs, and 30 healthy volunteers. However, for this analysis, we focused solely on dialysis patients, as there were no deaths or CV events in the control group and only one non-fatal CV event in the KTRs, leading to their exclusion from the analysis. Patients were enrolled at the University Clinical Center in Gdansk, Poland, between March and December 2021. All patients undergoing dialysis at our hospital were informed about the study by the center’s dietitian. KTRs were informed about the study by the supervising physician.

**Figure 1:**
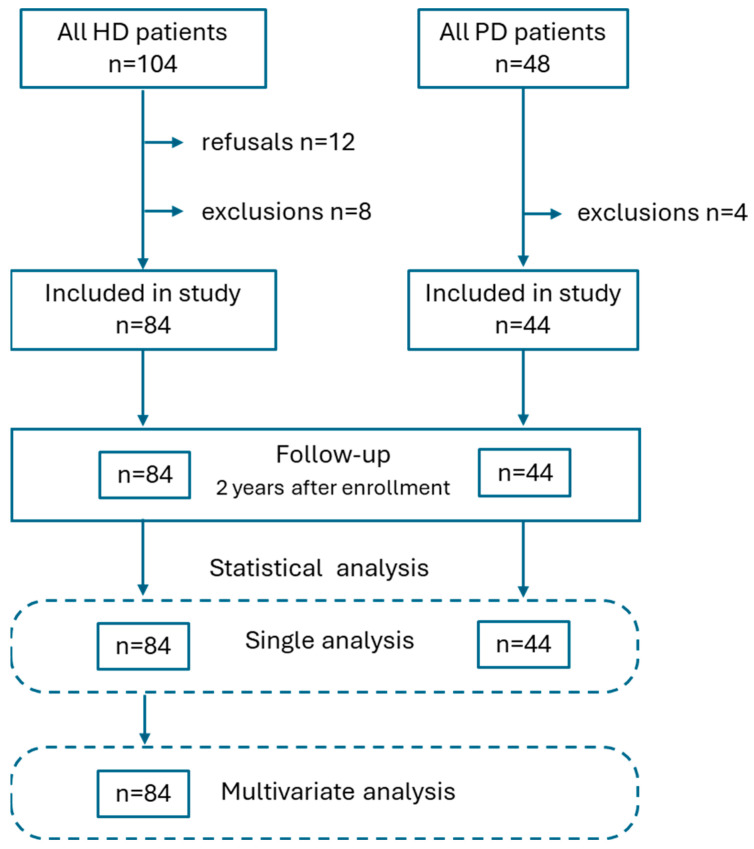
Study flowchart.

Two years after enrollment, a follow-up was conducted by reaching out to the patients or their families to evaluate their outcomes. This study’s flowchart is presented in Figure 1.

HD patients were treated with high-flux membranes (Helixone) with surface areas ranging from 1.4 to 2.2 m^2^. All patients underwent thrice-weekly HD sessions, typically lasting 4 h (3–4.5 h). For PD, patients on continuous ambulatory peritoneal dialysis (CAPD) performed 4 daily exchanges using a 2000–2500 mL low-glucose solution, while those on automated peritoneal dialysis (APD) received 10,000 mL of the low-glucose solution overnight. A more detailed description of the treatment can be found in the previous publication [12].

### 2.3. Assessment of Biochemical Data

For HD patients, blood samples were collected either after an overnight fast for those attending a morning dialysis session or after a 4 h fast for patients scheduled for dialysis in the afternoon or evening. For PD patients, blood was drawn in the morning following an overnight fast. Serum samples were stored at −80 °C until analysis. Adipokines, myokines, high-sensitivity *C*-reactive protein (hsCRP), and obestatin concentrations were measured using enzyme-linked immunosorbent assays (ELISA). The levels of TMAO, pCS, and IS were determined using liquid chromatography-tandem mass spectrometry (LC–MS/MS).

### 2.4. Assessment of Nutritional Status

Body composition was evaluated using a bioelectrical impedance analysis using a Body Composition Monitor (Fresenius Medical Care, Bad Homburg, Germany). Malnutrition was defined as a score of ≤5 on the 7-Point Subjective Global Assessment (SGA) [14]. Sarcopenia was characterized by reduced muscle strength and muscle mass [15], with low muscle strength defined as hand grip strength (HGS) below 16 kg for women and 27 kg for men [16]. Additionally, a lean tissue index (LTI) of less than 14 kg/m^2^ indicated low muscle mass [17].

### 2.5. Statistical Analysis

The statistical analyses were performed using Statistica 13.3 (StatSoft, Cracow, Poland), JASP 0.18.3, and Python version 3.10.12 in the Google Colab environment. The primary libraries used included lifelines, statsmodels, and Matplotlib 1.3.0 for creating the visualizations. Variables with a normal distribution were expressed as mean and standard deviation (SD), while those without a normal distribution were presented as medians with interquartile ranges (Q1–Q3). The distribution of variables was evaluated using histograms and the Shapiro–Wilk test. Group comparisons were conducted using either Student’s *t*-test or the Mann–Whitney U test, depending on the distribution of the variables. Categorical data were analyzed with the χ^2^ test or Fisher’s exact test. The survival was analyzed using Kaplan–Meier curves, and the differences between groups were evaluated using the log-rank test. In most cases, the cut-off points for the log-rank test (Mantel–Cox) were determined based on the median values. For body mass index (BMI), the cut-off was set at 25 kg/m^2^, for LTI at 14 kg/m^2^, and for potassium, phosphorus, albumin, and hsCRP, the upper limits of the reference ranges established in our laboratory were applied. The proportional hazards assumption was assessed using the Schoenfeld residuals test, with a *p*-value < 0.05 indicating a violation of the assumption. For variables that satisfied the proportional hazards assumption, a univariate Cox proportional hazards regression was performed. For variables that violated the assumption, an accelerated failure time (AFT) model with a log-normal distribution was employed to evaluate their influence on survival. We initially constructed models to examine the hypothesized relationships between adipokines, myokines, gut-microbiota-derived uremic toxins, nutritional status, and CV mortality, irrespective of *p*-values from the univariate analyses. These models were based on predefined research hypotheses. However, no significant associations were identified. Subsequently, variables with *p*-values < 0.1 in the univariate analyses were included in a multivariate model. Collinearity was assessed using the variance inflation factor (VIF), and only variables with VIF < 5 were retained in the final models. A significance level of *p* < 0.05 was applied for all tests.

## 3. Results

### 3.1. Patient Characteristics

The characteristics of the patients, divided into survivors (including patients who changed the method of KRTda) and those who died due to cardiovascular complications, are presented in Table 1. The analysis revealed that patients who died were significantly older in both the HD (71 vs. 60 years; *p* = 0.025) and PD (70 vs. 49 years; *p* = 0.029) groups. In the HD group, individuals who died from CV events had significantly lower blood phosphorus levels compared to survivors (4.0 vs. 5.4 mg/dL; *p* = 0.046). Conversely, in the PD group, those who passed away had a significantly higher BMI than survivors (32.4 vs. 26.2 kg/m^2^; *p* = 0.035).

In the HD group, 11.9% had diabetes type 1, 26.2% had diabetes type 2, and 91.7% had hypertension, while in the PD group, 13.6% had diabetes type 1, 22.7% had diabetes type 2, and 97.2% had hypertension. Before enrollment in the study, 22.6% of HD patients and 15.9% of PD patients had a myocardial infarction, and 3.6% of HD patients experienced a stroke. No significant differences in these conditions were observed between CV deaths and survivors in either group. There were also no significant differences between the prevalence of malnutrition and sarcopenia among the deceased and survivors.

### 3.2. Cardiovascular Mortality

**Figure 2:**
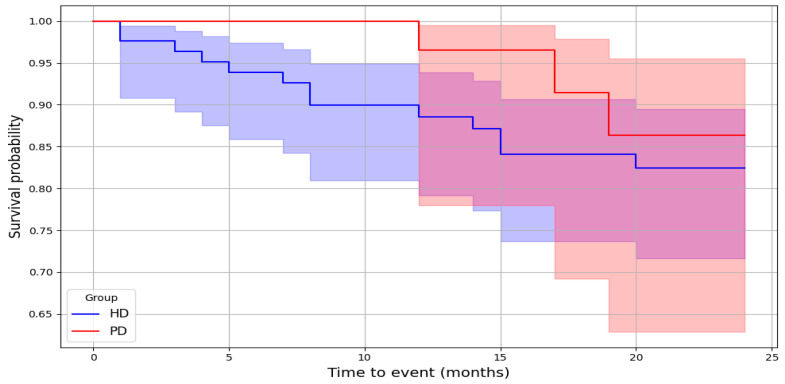
The Kaplan–Meier survival curves for cardiovascular mortality in HD and PD patients.

The mean follow-up was 18.2 (8) months in the HD group and 14.3 (8) months in the PD group, while the median follow-up was 24 months in the HD group and 13.5 months in the PD group. The Kaplan–Meier curves with 2-year CV mortality are presented in Figure 2.

**Figure 3:**
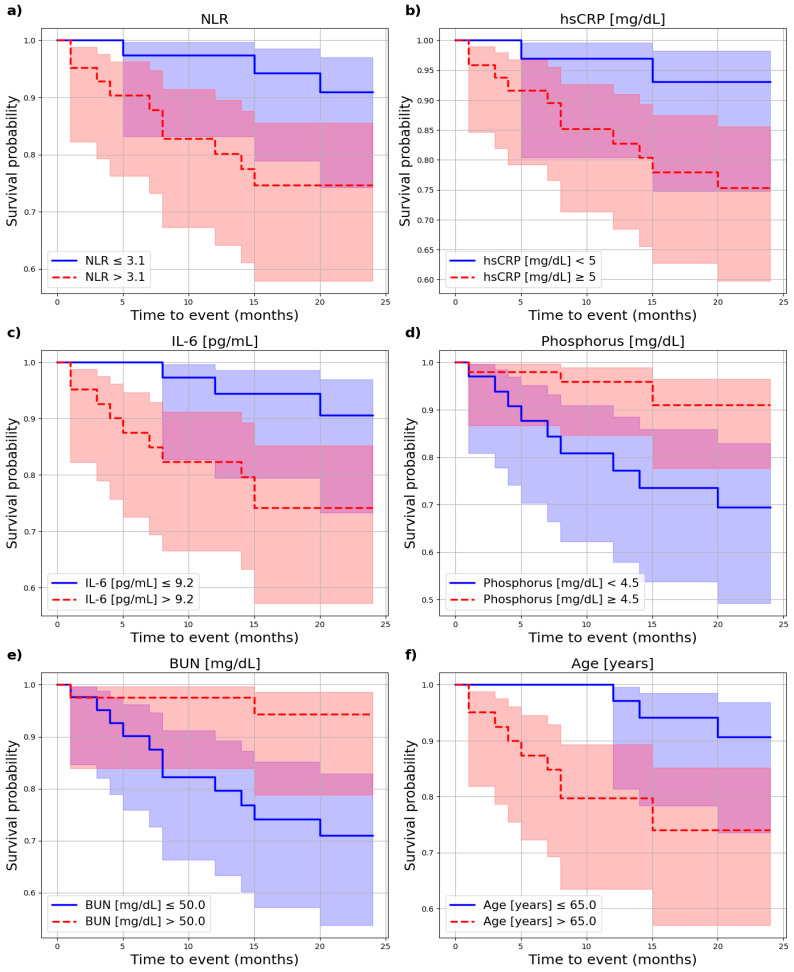
Impact of selected parameters on cardiovascular mortality in HD patients. (**a**) Impact of NLR on cardiovascular mortality in HD patients. (**b**) Impact of hsCRP on cardiovascular mortality in HD patients. (**c**) Impact of IL-6 on cardiovascular mortality in HD patients. (**d**) Impact of phosphorus on cardiovascular mortality in HD patients. (**e**) Impact of BUN on cardiovascular mortality in HD patients. (**f**) Impact of age on cardiovascular mortality in HD patients.

During this period, 15.5% of HD patients and 6.8% of PD patients died due to CVD. In total, 17.9% of HD patients and 27.3% of PD patients underwent kidney transplantation, while 7.1% of HD patients and 13.6% of PD patients died from non-CV causes (HD: sepsis n = 3, cancer n = 3; PD: sepsis n = 3, COVID-19 n = 3). Additionally, 34.1% of PD patients transitioned to HD during follow-up. The association of biochemical and nutritional parameters on CV mortality in the HD and PD groups was assessed. The results are presented in Table 2. In the HD group, significant associations with CV mortality were observed for age (*p* = 0.031), blood urea nitrogen (BUN) (*p* = 0.009), phosphorus (*p* = 0.013), interleukin-6 (IL-6) (*p* = 0.038), hsCRP (*p* = 0.046), and neutrophil-to-lymphocyte ratio (NLR) levels (*p* = 0.040), as illustrated in Figure 3.

**Figure 4:**
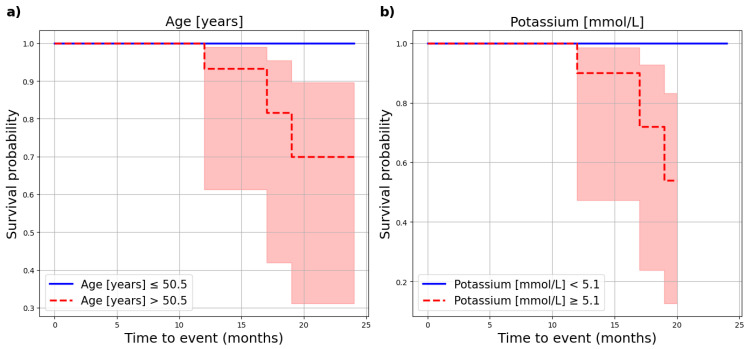
Impact of selected parameters on cardiovascular mortality in PD patients. (**a**) Impact of age on cardiovascular mortality in PD patients. (**b**) Impact of potassium on cardiovascular mortality in PD patients.

CV mortality was significantly associated with age (*p* = 0.049) and potassium levels (*p* = 0.005) in the PD group, as shown in Figure 4.

**Figure 5:**
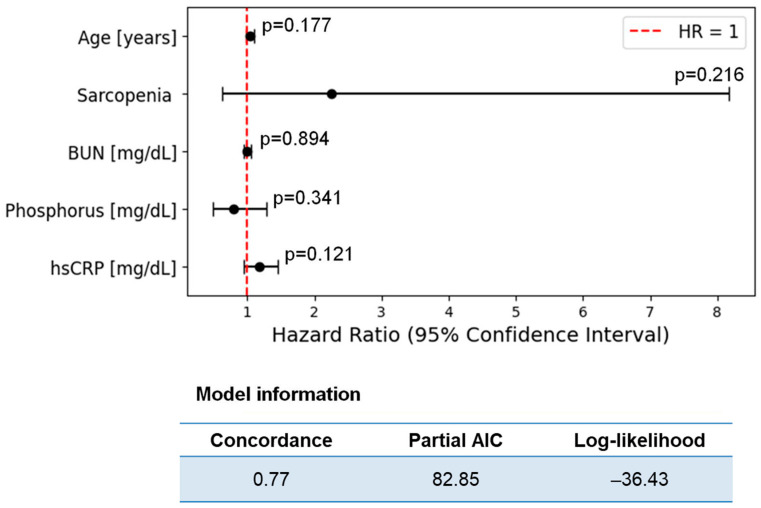
Forest plot of Hazard Ratios (HRs) with 95% confidence intervals for the multivariable Cox proportional hazards model, including model summary information.

Based on the univariate Cox regression analysis, age was identified as a significant factor influencing survival among both HD and PD patients. In HD patients, age showed a statistically significant result (Hazard Ratio—HR = 1.04, *p* = 0.040), indicating an increased risk of CV mortality with advancing age. For PD patients, both age (HR = 1.14, *p* = 0.041) and BUN concentration (HR = 1.1, *p* = 0.03) were significantly associated with survival (Table 3). A multivariable Cox proportional hazards model was developed to evaluate the association between clinical and biochemical parameters and the risk of CV mortality. The results are presented in Figure 5. None of the variables reached statistical significance (*p* > 0.05).

### 3.3. Cardiovascular Risk

During the 2-year follow-up, 9.5% of HD patients experienced a non-fatal CV event, whereas all CV events in PD patients were fatal. In the HD group, CV risk (both fatal and non-fatal CV events) was analyzed, revealing a significant association with age (*p* = 0.02) in the log-rank test, while IS concentration (*p* = 0.056) and myostatin/IL ratio (*p* = 0.088) showed a trend toward significance. Age was a significant predictor of CVR in the univariate Cox regression analysis (HR = 1.04, *p* = 0.008). Factors that approached statistical significance included BUN (HR = 0.97, *p* = 0.07) and obestatin (HR = 1.01, *p* = 0.08).

## 4. Discussion

This study aimed to explore the association between adipokines, myokines, gut-microbiota-derived uremic toxins, other biochemical factors, nutritional status, and their impact on 2-year CV mortality in patients undergoing KRT. Our univariate Cox regression analysis revealed that age was a significant predictor of CV mortality in the HD group, whereas both age and BUN levels were significant risk factors in the PD group. The log-rank analysis further identified specific risk factors in the dialysis subgroups. For HD patients, the key factors associated with higher CV mortality included age, BUN, the NLR, phosphorus levels, IL-6, and hsCRP. HD patients who died had significantly lower myostatin/IL-6 ratios. For PD patients, age and potassium levels emerged as the primary risk factors. We also attempted to develop multivariate models based on the available literature regarding the associations between the concentrations of the studied substances and survival. However, these models yielded non-significant results and demonstrated a poor fit to the data. Given these limitations, we constructed a multivariate Cox model based on the variables identified in the univariate analysis (with *p* < 0.1)—age, sarcopenia, BUN, phosphorus, and hsCRP—traditional variables that are characteristic of patients with CKD undergoing KRT. Despite the lack of statistical significance, certain variables in the multivariate Cox analysis showed trends toward increased CV mortality risk, specifically age, sarcopenia, and hsCRP, while phosphorus seemed to trend toward a decreased risk. The trends observed in this study, particularly with the myostatin/IL-6 and irisin/IL-6 ratios, require further investigation. A deeper understanding of the complex interplay between inflammation, muscle health, and metabolic dysfunction in CKD patients could lead to the development of improved prognostic tools and more effective interventions aimed at reducing CV mortality.

### 4.1. Adipokines

In our study, among the adipokines analyzed, only IL-6 showed a significant effect on the survival of HD patients, as determined using the log-rank test. Patients with elevated IL-6 levels had significantly poorer survival outcomes compared to those with lower IL-6 levels. This finding highlights the role of IL-6 as a biomarker of inflammation and its negative impact on prognosis in HD patients. Elevated IL-6 levels may indicate a state of chronic inflammation, which is well-documented to contribute to adverse cardiovascular outcomes and increased overall mortality in dialysis patients [18,19]. Another inflammatory parameter that significantly impacted CV mortality in our HD group was hsCRP. Patients with hsCRP levels ≥ 5 mg/dL had significantly higher mortality rates. This association has also been reported in other studies involving HD and PD patients [20,21]. In our study, no significant effect of leptin, adiponectin, or ZAG on CV mortality or risk was observed, although their impact on it has been reported in other studies. In a cohort of patients with CKD stages 3–4, Menon et al. found that higher adiponectin levels were associated with an increased risk of both all-cause and cardiovascular (CVD) mortality [22]. A similar relationship was observed in HD patients, where elevated adiponectin levels were linked to a threefold higher risk of mortality [23]. The leptin/adiponectin ratio (LAR) is recognized as a marker of CVD. Elevated LAR values are strongly associated with increased CVR in the general population [24]. However, findings among dialysis patients remain inconclusive and require further investigation [25]. In our study, we did not observe statistically significant results regarding the impact of LAR on CV mortality. We also evaluated other adipokine and myokine ratios. HD patients who died had a significantly lower myostatin/IL-6 ratio compared to survivors, indicating a link between inflammation, muscle health, and CV mortality. This association was statistically significant under the log-normal model (*p* = 0.044). In the log-rank analysis among PD patients, the irisin/IL-6 ratio was nearly a significant factor influencing CV mortality (*p* = 0.05). To the best of our knowledge, this is the first study to investigate these specific ratios. This novel finding suggests that the myostatin/IL-6 and irisin/IL-6 ratio could serve as a dual marker of inflammation and muscle health, highlighting its potential utility in identifying patients at high risk of CVD In the study by Bouchara et al., a significant correlation was observed between serum ZAG concentrations, and both all-cause mortality and cardiovascular events [26].

### 4.2. Myokines

Myokines are a group of cytokines and polypeptides released by skeletal muscles. Their altered concentrations in patients with CKD may be associated with the development and progression of CVD [27]. According to Sakashita et al., myostatin concentrations at the initiation of dialysis can predict mortality or hospitalization within one year of starting dialysis. The authors explain that this association between myostatin levels and prognosis may be linked with sarcopenia, frailty, and poor nutritional status [28]. Irisin production declines during CKD, likely due to sarcopenia and reduced physical activity. Arcidiacono et al. found that patients with the highest serum irisin levels had a lower CVR compared to those with lower levels, suggesting its potential as a marker for CV outcomes in CKD [29]. Dong et al. confirmed that lower irisin levels are associated with increased cardiovascular mortality in hemodialysis patients [30]. In our study, no significant effect of myostatin or irisin on CV mortality or risk was observed.

### 4.3. Gut-Microbiota-Derived Uremic Toxins

Metabolites derived from gut microbiota play a crucial role in increasing CVR in patients with CKD. TMAO is strongly linked to CVD by driving vascular inflammation, endothelial dysfunction, and vascular calcification through macrophage recruitment, oxidative stress, and the activation of inflammatory pathways in endothelial cells. PCS intensifies vascular inflammation, induces CD4 + T-cell apoptosis, and disrupts immune function. Similarly, IS contributes to oxidative stress and inflammation, resulting in endothelial dysfunction, atherosclerosis, and reduced proliferation of CD4 + T-cells [31]. In the study by Shafi et al., higher TMAO levels were associated with an increased risk of CV mortality in HD patients, particularly among Caucasians. In this group, a twofold increase in TMAO levels was linked to a higher risk of cardiac death and all-cause mortality. Among African American patients, the association was nonlinear and significant only for cardiac deaths in patients with TMAO levels below the median [32]. In the study by Zhang et al., high plasma TMAO levels were also found to be independently associated with increased CV and all-cause mortality in HD patients [33]. In contrast, other researchers found no significant associations between TMAO levels and CV or all-cause mortality and CV events [34,35]. Zhao et al., in a letter to *Renal Failure*, explain that the inconsistency of study results may partly be attributed to factors such as measurement methods, dialysis modes, residual renal function, intestinal microbiota, diet, and the precise definition of high and low TMAO levels [36], which can be applied not only to TMAO, but also to other studied metabolites. In our study, we also observed no association between gut-microbiota-derived uremic toxins and CV mortality. Potential confounding factors such as dietary protein intake, antibiotic use, and residual renal function could have influenced the gut microbiota composition and uremic toxin levels. Variations in dietary protein could have altered the composition and functions of the gut microbiota, potentially affecting the levels of uremic toxins. Antibiotics could have reduced beneficial bacteria that typically regulate uremic toxin production. Additionally, preserved residual renal function might have lower levels of uremic toxins due to better toxin clearance. All of these factors could have masked the effects of uremic toxins on CV outcomes. In the case of CVR, a result approaching statistical significance was observed for IS (*p* = 0.056). In a meta-analysis, Lin et al. reported that higher concentrations of pCS and IS are linked to increased mortality in CKD patients, while pCS alone, unlike IS, is associated with a greater CVR [37]. In the study conducted by Li et al., IS was found to be associated with CV mortality among HD patients [38].

### 4.4. Other Biochemical Parameters

Higher serum phosphate levels have been linked to an increased risk of CVD events in numerous studies, both in the general population and among patients with CKD. Hyperphosphatemia is strongly associated with vascular calcification and may contribute to CVD risk by inhibiting vitamin D activation [39]. However, some studies have observed that individuals with higher serum phosphorus concentrations had better prognosis. For example, in the study by Sun et al., lower serum phosphorus concentrations in HD patients were associated with a higher risk of mortality [40]. Similarly, in our study, lower phosphorus levels were associated with CV mortality among HD patients. Notably, in our study, none of the participants had phosphorus concentrations below the normal range.

Therefore, the association between lower phosphorus levels and higher mortality cannot be attributed to malnutrition or the accompanying lower phosphorus concentrations often linked to poor outcomes. Also, surprisingly, we observed, in our study, that HD patients with higher BUN had a lower risk of CV death. The NLR is increasingly being investigated as a novel and easily accessible inflammatory marker. Elevated NLR has been associated with systemic inflammation and poor outcomes in various chronic diseases. Among patients with CKD, a higher NLR is linked to increased proteinuria, elevated serum creatinine levels, and reduced estimated glomerular filtration rate (eGFR) [41]. In our study, HD patients with higher NLRs were found to have greater CV mortality, consistent with findings from the study by Neuen et al., which demonstrated a strong association between elevated NLR and adverse CV outcomes [42]. This highlights the potential of NLR as a prognostic marker for CVR in the HD population. Given its simplicity and cost-effectiveness, NLR could be a valuable tool in clinical practice to identify high-risk patients.

We observed that, in the PD group, patients with potassium levels > 5.1 mmol/L had a higher risk of death. However, it should be taken into account that this was only a single measurement and a small group of patients. In a cohort study by Torlen et al., a U-shaped association was observed in PD patients: both low (<3.5 mmol/L) and high (≥5.5 mmol/L) potassium levels were linked to increased all-cause and CV mortality [43]. In our HD group, obestatin, a ghrelin-associated peptide, was close to a statistically significant result in the Cox analysis of CV risk (HR = 1.01, *p* = 0.08). According to Beberashvili et al., low serum obestatin concentration is an independent predictor of mortality in HD patients [44].

The limitations of our study are the small sample size and the low number of events, which likely contributed to the lack of significant findings. Traditional factors may overshadow the effects of non-traditional ones, making their influence difficult to detect in studies with smaller cohorts. Another limitation of this study was that blood samples were collected only before dialysis. Analyzing metabolite concentrations after dialysis could have provided more precise and comprehensive insights. Null findings regarding the impact of the studied compounds on cardiovascular mortality may indicate the absence of a direct relationship, but they also emphasize the need to account for potential confounding factors and study design limitations. These results suggest that further research is needed to better understand the underlying mechanisms and refine our approach to exploring factors influencing cardiovascular survival.

Future research should focus on exploring the complex interplay between gut-microbiota-derived metabolites, adipokines, myokines, and nutritional status in shaping CVR in CKD. Understanding these interactions is essential to uncover their collective impact on CVD progression. A large-scale study measuring these biomarkers in a well-characterized cohort would provide invaluable insights, but may face challenges due to cost and logistical constraints. Alternatively, pooling data from published studies with well- matched methodologies or conducting multicenter investigations could enhance the reliability of the findings. These efforts would not only clarify the role of these biomarkers in CV mortality, but also open new avenues for developing targeted therapeutic and nutritional interventions for patients with CKD.

## 5. Conclusions

The studied adipokines, myokines, and gut-microbiota-derived uremic toxins have a weaker direct impact on survival compared to well-established markers such as age, nutritional status, and inflammatory factors. However, the irisin/IL-6 and myostatin/IL-6 ratios emerged as potentially important indicators, suggesting a link between inflammation, muscle health, and CV mortality. Further research involving a larger patient population is essential to validate these findings and confirm their clinical relevance.

## Figures and Tables

**Table 1:** Characteristics of HD and PD survivors and patients who died from cardiovascular complications during the 2-year follow-up.

	HD Patients (N = 84)			PD Patients (N = 44)		
	CV Deaths During Follow-Up	Survivors	*p*-Values	CV Deaths During Follow-Up	Survivors	*p*-Values
N	13	71	*p*-values	3	41	*p*-values
Males, *n* (%)	9 (69.2)	39 (54.9)	0.33	3 (100)	20 (48.8)	0.04
Age (years)	71.0 (12.4)	60.0 (16.6)	0.025	70.0 [70.0–79.0]	49.0 [36.0–66.0]	0.029
Dialysis vintage (months)	27.0 [8.0–95.0]	30.0 [9.5–71.0]	0.643	12.0 [9.5–38.0]	14.5 [8.5–25.2]	0.886
BMI (kg/m^2^)	23.1 [22.1–28.6]	24.8 [22.5–27.6]	0.961	32.4 (3.1)	26.2 (4.9)	0.035
LTI (kg/m^2^)	10.8 (2.4)	11.7 (2.7)	0.309	15.5 [14.5–16.5]	13.1 [11.1–14.7]	0.183
BUN (mg/L)	45.5 (15.0)	53.5 (13.9)	0.06	63.0 (18.7)	48.4 (13.6)	0.087
HCT (%)	33.1 (4.3)	31.3 (3.8)	0.126	33.2 (2.7)	33.6 (4.8)	0.879
Hb (g/dL)	10.7 (1.4)	10.3 (1.2)	0.288	11.1 (1.0)	11.4 (1.7)	0.767
NLR	3.5 [3.2–3.9]	3.0 [2.1–4.4]	0.19	3.3 (1.4)	3.8 (1.6)	0.585
PLR	157.4 [127.7–192.4]	138.7 [101.5–190.3]	0.466	193.4 (64.5)	192.6 (69.5)	0.983
Potassium (mmol/L)	5.3 (0.5)	5.3 (0.8)	0.858	5.3 [5.2–5.3]	4.4 [4.1–5.0]	0.084
Calcium (mg/dL)	8.7 (0.6)	8.7 (0.9)	0.922	8.6 (0.1)	8.8 (0.7)	0.649
Phosphorus (mg/dL)	4.0 [3.3–4.6]	5.4 [4.0–6.5]	0.046	6.3 (0.6)	5.8 (1.8)	0.686
Albumin (g/dL)	3.25 [3.1–3.42]	3.4 [3.2–3.60]	0.191	32.0 [31.5–34.0]	34.0 [29.0–37.0]	0.797
TMAO (μM/L)	109.4 [95.6–168.4]	124.9 [76.3–203.4]	0.855	119 [115.7–149.2]	117.9 [78.4–196.1]	0.759
pCS (μM/L)	170.4 (95.8)	193.2 (85.1)	0.426	169.8 (83.1)	146.3 (94.8)	0.679
(IS μM/L)	33.9 [28.4–47.1]	43.5 [30.3–64.7]	0.225	38.6 [17.1–53.6]	34.7 [15.9–33.2]	0.852
leptin (ng/mL)	5.9 [1.8–14.6]	7.6 [4.1–19.9]	0.335	13.5 [11.8–23.1]	14.1 [5.3–36.1]	0.861
adiponectin (μg/mL)	4.8 [2.8–9.3]	5.3 [2.7–9.3]	0.678	4.8 (4.6)	8.5 (4.9)	0.21
ZAG (μg/mL)	11.7 [10.7–15.9]	11.0 [8.4–14.3]	0.367	12.8 [9.9–39.1]	10.7 [8.5–15.0]	0.627
IL-6 (pg/mL)	12.6 [11.0–14.7]	8.2 [5.0–18.2]	0.107	7.9 [7.4–11.4]	5.7 [2.8–13.7]	0.328
irisin (μg/mL)	7.6 [6.8–8.1]	7.2 [6.1–8.4]	0.496	7.8 [6.8–8.1]	9.3 [8.1–10.2]	0.061
myostatin (pg/mL)	2816.8 [1698.4–4000.0]	3482.4 [2356.0–4710.0]	0.182	5214.9 (2503.6)	6735.1 (2753.8)	0.359
LAR	0.8 [0.3–5.9]	1.7 [0.5–7.4]	0.282	10.7 [5.8–10.7]	2.7 [0.6–6.1]	0.394
irisin/IL-6	0.6 [0.5–0.8]	0.7 [0.4–1.5]	0.266	0.9 [0.7–1.0]	1.8 [0.7–3.5]	0.258
myostatin/IL-6	215.9 [115.5–392.0]	352.2 [194.1–702.3]	0.031	765.4 [473.7–872.3]	831.4 [508.2–2804.2]	0.421
hsCRP (mg/dL)	9.8 [7.5–10.0]	6.7 [3.0–10.0]	0.176	9.2 [8.8–9.5]	3.3 [1.9–9.8]	0.258
obestatin (pg/mL)	158.0 [157.0–575.0]	160.0 [154.0–425.0]	0.872	154.0 [119.0–154.5]	155.0 [87.0–488.0]	0.402
ADMA (μM/L)	1.0 [0.7–1.3]	0.8 [0.5–1.3]	0.328	0.9 [0.6–1.2]	0.6 [0.4–1.0]	0.816

CV, cardiovascular; BMI, body mass index; LTI, lean tissue index; BUN, blood urea nitrogen; HCT, hematocrit; Hb, hemoglobin; NLR, neutrophil-to-lymphocyte ratio; PLR, platelet-to-lymphocyte ratio; TMAO, trimethylamine-*N*-oxide; pCS, *p*-cresyl sulfate; IS, indoxyl sulfate; ZAG, zinc alpha 2-glycoprotein; IL-6, interleukin 6; LAR, leptin/adiponectin ratio; hsCRP, high-sensitivity *C*-reactive protein; ADMA, asymmetric dimethylarginine.

**Table 2:** The log-rank test results assessing the association of biochemical and nutritional parameters with cardiovascular mortality in HD and PD patients.

	HD Patients			PD Patients		
	Cut-Off Points	Log-Rank	*p*-Values	Cut-Off Points	Log-Rank	*p*-Values
Gender [0-W; 1-M]	binary	1.0	0.321	binary	3.3	0.070
Age (years)	65.0	4.7	0.031	50.5	3.8	0.049
Dialysis vintage (months)	28.5	0.4	0.540	14.0	0.4	0.527
BMI (kg/m^2^) *	25.0	0.1	0.822	25.0	2.5	0.114
malnutrition [SGA ≤ 5]	binary	0.1	0.748	0.0	1.9	0.165
sarcopenia	binary	3.0	0.081	0.0	1.3	0.250
LTI (kg/m^2^) *	14.0	0.3	0.565	14.0	0.8	0.375
BUN (mg/L)	50.0	6.8	0.009	49.5	2.5	0.114
HCT (%)	31.4	2.2	0.140	33.2	0.1	0.715
Hb (g/dL)	10.3	0.3	0.613	11.1	0.1	0.715
NLR	3.1	4.2	0.040	3.8	0.6	0.449
PLR	142.2	0.6	0.453	190.6	0.5	0.488
Potassium (mmol/L) *	5.1	0.7	0.388	5.1	7.9	0.005
Calcium (mg/dL)	8.8	0.9	0.354	8.8	3.1	0.076
Phosphorus (mg/dL) *	4.5	6.2	0.013	4.5	1.3	0.259
Albumin (g/dL) *	35.0	1.0	0.321	35.0	0.2	0.686
TMAO (μM/L)	124.5	0.1	0.727	118.2	0.7	0.408
pCS (μM/L)	172.6	0.1	0.821	143.3	0.5	0.488
(IS μM/L)	41.8	1.8	0.177	36.7	1.1	0.290
leptin (ng/mL)	7.2	1.2	0.279	13.8	0.3	0.614
adiponectin (μg/mL)	5.3	0.2	0.694	7.7	0.2	0.686
ZAG (μg/mL)	11.5	1.0	0.315	10.9	0.3	0.598
IL-6 (pg/mL)	9.2	4.3	0.038	5.8	3.3	0.070
irisin (μg/mL)	7.2	0.0	0.826	9.1	2.2	0.135
myostatin (pg/mL)	3333.8	0.7	0.395	6418.0	0.2	0.625
leptin/adiponectin	1.57	1.14	0.28	2.65	0.04	0.84
irisin/IL-6	0.69	1.09	0.30	1.4	3.83	0.05
myostatin/IL-6	320.1	0.89	0.34	771.1	0.43	0.51
hsCRP (mg/dL) *	5.0	4.0	0.046	5.0	3.4	0.064
obestatin (pg/mL)	159.5	0.9	0.343	155.0	2.1	0.144
ADMA (μM/L)	0.9	1.3	0.263	0.7	0.2	0.625

BMI, body mass index; LTI, lean tissue index; BUN, blood urea nitrogen; HCT, hematocrit; Hb, hemoglobin; NLR, neutrophil-to-lymphocyte ratio; PLR, platelet-to-lymphocyte ratio; TMAO, trimethylamine-*N*-oxide; pCS, *p*-cresyl sulfate; IS, indoxyl sulfate; ZAG, zinc alpha 2-glycoprotein; IL-6, interleukin 6; hsCRP, high-sensitivity *C*-reactive protein; ADMA, asymmetric dimethylarginine. * Cut-off points are the upper limits of reference values.

**Table 3:** Univariate Cox regression analysis of factors associated with cardiovascular mortality in HD and PD patients.

	HD Patients				PD Patients			
	Hazard Ratio (HR)	z	*p*-Values	Concordance	Hazard Ratio (HR)	z	*p*-Values	Concordance
Gender [0-W; 1-M]	1.80	0.98	0.327	0.57	complete separation—excluded from analysis			
Age (years)	1.04	2.05	0.040	0.69	1.14	2.05	0.041	0.90
Dialysis vintage (months)	-	-	-	-	1.00	0.00	0.998	0.41
BMI (kg/m^2^)	1.01	0.20	0.842	0.51	1.26	1.83	0.068	0.81
malnutrition [SGA ≤ 5]	1.20	0.32	0.747	0.53	0.00	−0.01	0.996	0.71
sarcopenia	2.93	1.66	0.096	0.65	4.43	1.05	0.293	0.61
LTI (kg/m^2^)	0.91	−0.73	0.468	0.56	1.60	1.50	0.134	0.88
BUN (mg/L)	0.96	−1.91	0.057	0.66	1.10	2.18	0.030	0.82
HCT (%)	1.11	1.45	0.146	0.60	0.95	−0.41	0.684	0.53
Hb (g/dL)	-	-	-	-	0.84	−0.46	0.646	0.56
NLR	1.03	0.66	0.510	0.61	0.80	−0.54	0.589	0.65
PLR	1.00	0.45	0.652	0.56	1.00	0.19	0.846	0.60
Potassium (mmol/L)	1.09	0.22	0.823	0.55	3.68	1.63	0.104	0.80
Calcium (mg/dL)	0.99	−0.03	0.979	0.52	0.48	−0.75	0.451	0.61
Phosphorus (mg/dL)	0.70	−1.93	0.053	0.68	1.21	0.67	0.504	0.60
Albumin (g/dL)	0.93	−1.17	0.243	0.64	1.02	0.18	0.856	0.51
TMAO (μM/L)	1.00	−0.57	0.566	0.51	1.00	0.05	0.963	0.63
pCS (μM/L)	1.00	0.76	0.449	0.54	1.00	0.69	0.487	0.57
(IS μM/L)	0.99	−1.01	0.312	0.59	1.00	−0.02	0.984	0.49
leptin (ng/mL)	0.98	−1.10	0.272	0.60	0.99	−0.38	0.703	0.51
adiponectin (μg/mL)	1.00	−0.04	0.966	0.49	0.83	−1.13	0.260	0.67
ZAG (μg/mL)	0.99	−0.28	0.782	0.39	1.05	1.94	0.053	0.52
IL−6 (pg/mL)	1.06	1.22	0.222	0.65	1.06	0.70	0.484	0.70
irisin (μg/mL)	1.08	0.52	0.603	0.54	0.54	−1.45	0.147	0.76
myostatin (pg/mL)	1.00	−1.40	0.161	0.63	1.00	−1.03	0.301	0.63
LAR	0.96	−0.8	0.42	0.59	1.1	0.94	0.35	0.6
irisin/IL-6	0.41	−1.52	0.13	0.61	0.35	−1.1	0.27	0.7
myostatin/IL-6	-	-	-	-	1	−0.96	0.34	0.63
hsCRP (mg/dL)	1.18	1.68	0.094	0.60	1.43	1.35	0.177	0.73
obestatin (pg/mL)	1.00	0.20	0.844	0.52	1.00	−0.73	0.468	0.64
ADMA (μM/L)	1.03	0.08	0.939	0.60	2.04	0.45	0.654	0.51
Variables that do not meet the assumptions of proportionality of distributions; Log-Normal model								
	exp (coef)	z	*p*-values	Concordance index	-	-	-	-
Dialysis vintage (months)	0.99	−0.80	0.422	0.51	-	-	-	-
Hb (g/dL)	0.82	−0.67	0.504	0.56	-	-	-	-
myostatin/IL-6	1.0039	2.01	0.044	0.7	-	-	-	-

BMI, body mass index; LTI, lean tissue index; BUN, blood urea nitrogen; HCT, hematocrit; Hb, hemoglobin; NLR, neutrophil-to-lymphocyte ratio; PLR, platelet-to-lymphocyte ratio; TMAO, trimethylamine-*N*-oxide; pCS, *p*-cresyl sulfate; IS, indoxyl sulfate; ZAG, zinc alpha 2-glycoprotein; IL-6, interleukin 6; LAR, leptin/adiponectin ratio; hsCRP, high-sensitivity *C*-reactive protein; ADMA, asymmetric dimethylarginine.

## Data Availability

The data sets used and/or analyzed during the current study are available from the corresponding author upon reasonable request.

## Abbreviations

ADMA	asymmetric dimethylarginine
APD	automated peritoneal dialysis
BMI	body mass index
BUN	blood urea nitrogen
CAPD	continuous ambulatory peritoneal dialysis
CKD	chronic kidney disease
CV	cardiovascular
CVD	cardiovascular diseases
ELISA	enzyme-linked immunosorbent assays
Hb	hemoglobin
HCT	hematocrit
HD	hemodialysis
HGS	hand grip strength
HR	hazard risk
hsCRP	high sensitivity *C*-reactive protein
IL-6	interleukin 6
IS	indoxyl sulfate
KRT	kidney replacement therapy
KTRs	kidney transplant recipients
LAR	leptin/adiponectin ratio
LC─MS/MS	liquid chromatography-tandem mass spectrometry
LTI	lean tissue index
NLR	neutrophil-to-lymphocyte ratio
pCS	*p*-cresyl sulfate
PD	peritoneal dialysis
PLR	platelet-to-lymphocyte ratio
ROS	reactive oxygen species
SGA	7-Point Subjective Global Assessment
TMAO	trimethylamine-*N*-oxide
ZAG	zinc alpha 2-glycoprotein
